# DNAM-1 and the TIGIT/PVRIG/TACTILE Axis: Novel Immune Checkpoints for Natural Killer Cell-Based Cancer Immunotherapy

**DOI:** 10.3390/cancers11060877

**Published:** 2019-06-23

**Authors:** Beatriz Sanchez-Correa, Isabel Valhondo, Fakhri Hassouneh, Nelson Lopez-Sejas, Alejandra Pera, Juan M. Bergua, Maria Jose Arcos, Helena Bañas, Ignacio Casas-Avilés, Esther Durán, Corona Alonso, Rafael Solana, Raquel Tarazona

**Affiliations:** 1 Immunology Unit, Department of Physiology, University of Extremadura, 10003 Cáceres, Spain; beatrizsanchezcorrea@gmail.com (B.S.-C.); ivalhondog@gmail.com (I.V.); hassounehfakhri@yahoo.com (F.H.); nelsonj836@hotmail.com (N.L.-S.); 2 Instituto Maimónides de Investigación Biomédica de Córdoba (IMIBIC), 14004 Córdoba, Spain; alejandrapera@gmail.com (A.P.); corona_alonso@hotmail.com (C.A.); 3 Department of Cell Biology, Physiology and Immunology, University of Córdoba, 14004 Córdoba, Spain; 4 Department of Hematology, Hospital San Pedro de Alcantara, 10003 Cáceres, Spain; jmbergua@icloud.com (J.M.B.); mjarcar@yahoo.es (M.J.A.); cromacita@gmail.com (H.B.); nachocasas@hotmail.com (I.C.-A.); 5 Histology and Pathology Unit, Faculty of Veterinary, University of Extremadura, 10003 Cáceres, Spain; esther@unex.es; 6 Reina Sofia University Hospital, 14004 Córdoba, Spain

**Keywords:** NK cells, cancer immunotherapy, CD155, CD112, DNAM-1, PVRIG, TACTILE, TIGIT

## Abstract

Natural killer (NK) cells are lymphocytes of the innate immune response characterized by their role in the destruction of tumor cells. Activation of NK cells depend on a fine balance between activating and inhibitory signals mediated by different receptors. In recent years, a family of paired receptors that interact with ligands of the Nectin/Nectin-like (Necl) family has attracted great interest. Two of these ligands, Necl-5 (usually termed CD155 or PVR) and Nectin-2 (CD112), frequently expressed on different types of tumor cells, are recognized by a group of receptors expressed on T and NK cells that exert opposite functions after interacting with their ligands. These receptors include DNAM-1 (CD226), TIGIT, TACTILE (CD96) and the recently described PVRIG. Whereas activation through DNAM-1 after recognition of CD155 or CD112 enhances NK cell-mediated cytotoxicity against a wide range of tumor cells, TIGIT recognition of these ligands exerts an inhibitory effect on NK cells by diminishing IFN-γ production, as well as NK cell-mediated cytotoxicity. PVRIG has also been identified as an inhibitory receptor that recognizes CD112 but not CD155. However, little is known about the role of TACTILE as modulator of immune responses in humans. TACTILE control of tumor growth and metastases has been reported in murine models, and it has been suggested that it negatively regulates the anti-tumor functions mediated by DNAM-1. In NK cells from patients with solid cancer and leukemia, it has been observed a decreased expression of DNAM-1 that may shift the balance in favor to the inhibitory receptors TIGIT or PVRIG, further contributing to the diminished NK cell-mediated cytotoxic capacity observed in these patients. Analysis of DNAM-1, TIGIT, TACTILE and PVRIG on human NK cells from solid cancer or leukemia patients will clarify the role of these receptors in cancer surveillance. Overall, it can be speculated that in cancer patients the TIGIT/PVRIG pathways are upregulated and represent novel targets for checkpoint blockade immunotherapy.

## 1. Introduction

The immune system response to pathogens is controlled by different regulatory mechanisms to maintain tolerance to self and protect tissue integrity. Several signaling pathways mediated by inhibitory receptors have been described to contribute to immune homeostasis while defending against infected and transformed cells. Among these inhibitory receptors, cytotoxic T-lymphocyte-associated protein 4 (CTLA-4) and programmed cell death protein 1 (PD-1) have been well characterized in T cells and have been shown to play an important role in regulating T cell activation and effector functions, particularly in the context of cancer immunology [1]. The use of monoclonal antibodies (mAbs) targeting CTLA-4 or PD-1 checkpoint pathways have been approved for clinical use leading to durable clinical responses in various cancer types [2,3,4]. CTLA-4 is a co-inhibitory receptor that shares the same ligands (B7 family that includes CD80 and CD86) with CD28, the main T cell co-stimulatory signal, constituting the first evidence for paired activating-inhibitory receptors on T cells interacting with the same ligands expressed on other cell types [5]. Thus, inhibitory signals provided by an ample array of receptors are essential for immune homeostasis and tolerance of both T cells and natural killer (NK) cells. However, inhibitory signals also contribute to the immunosuppressive microenvironment in cancer and are preferred targets for cancer immunotherapy since checkpoint blockade therapy has been particularly successful in some cancer settings such as melanoma.

CD28 and CTLA-4 are paired receptors that, by interacting with B7 family ligands, regulate T cell activation but are not involved in the regulation of human NK cell function. Other two major families of paired co-stimulatory and inhibitory receptors that regulate NK cell function have been defined in humans. (i) The MHC class I-specific receptors such as Killer Immunoglobulin-like receptors (KIR) and NKG2 families [6] that include activating and inhibitory forms, and (ii) a group of receptors that interact with molecules of the Nectins and Nectin-like (Necls) family [7] and that include the activating receptor DNAM-1 (DNAX-associated molecule 1) and the inhibitory receptors TIGIT (T-cell immunoglobulin and ITIM domain), PVRIG (PVR-related Ig domain) and TACTILE (T cell activation, increased late expression), that constitute the TIGIT/PVRIG/TACTILE inhibitory axis involved in the control of NK cell function.

NK cells are innate lymphoid cells (ILC) playing major roles in the defense against tumors and virus-infected cells. NK cell constitutive expression of lytic proteins makes them ready-to-lyse target cells. NK cells recognize transformed cells that have lost the expression of major histocompatibility complex (MHC) antigens. In humans, peripheral blood NK cells can be classified into different subsets according to their surface receptor expression and functionality [8,9]. In addition, activated NK cells release interferon (IFN)-γ and tumor necrosis factor (TNF)-α that are involved in the destruction of target cells and promote inflammatory responses [10]. NK cell ability to lyse transformed cells without antigen-specificity makes them unique candidates for cancer treatment. NK cell function depends on a complex balance between signals transmitted through activating receptors and inhibitory receptors.

The major NK cell inhibitory receptors KIR and NKG2A recognize human leukocyte antigens (HLA) class I molecules and prevent NK cell-mediated lysis of healthy cells [11]. HLA class I loss is a frequent event on tumor-transformed cells and virus-infected cells that increases their susceptibility to NK cell-mediated lysis, whereas cancer cells expressing HLA class I molecules are resistant to NK cell-mediated lysis. The anti-leukemia role of NK cells is supported by studies on haemopoietic stem cell transplantation showing that alloreactive NK cells (KIR mismatched) derived from haploidentical donors kill leukemia blasts preventing leukemia relapse [12,13]. NK cells are the first lymphocytes to appear after haemopoietic stem cell transplantation but frequently are dysfunctional compared to NK cells from healthy donors. Different strategies have been designed for NK cell manipulation including the use of checkpoint inhibitors [14]. Many studies have reported downregulation of activating receptors in peripheral blood NK cells from patients with hematological malignancies and solid cancers that correlated with disease progression. Despite the NK cells limited ability to infiltrate solid tumors and reduced cytotoxicity, accumulated evidence shows a role for NK cells in the control of metastasis [15,16]. However, little is known concerning infiltrating NK cells in solid tumor and the immunotherapeutic approaches designed to enhance NK cell activity in solid tumors [17].

**Figure 1:**
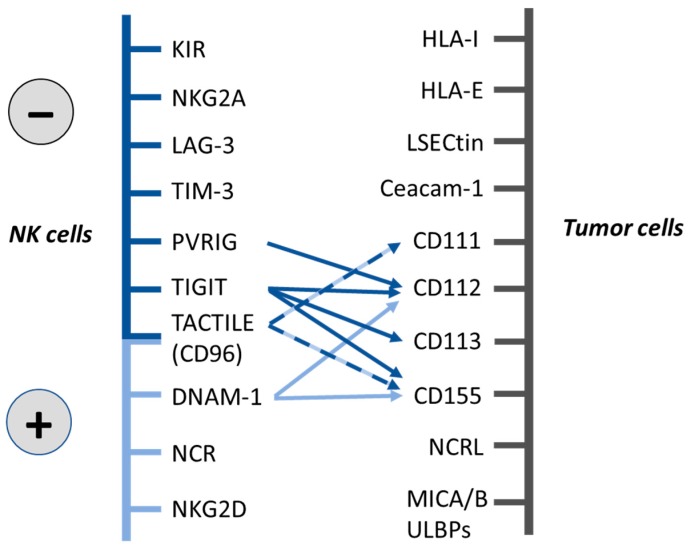
Natural killer (NK) cell activating and inhibitory receptors and their ligands on tumor cells. Inhibitory receptors are indicated in dark blue and activating receptors in light blue. While mouse TACTILE expresses only intracytoplasmic inhibitory motifs, human TACTILE expresses both inhibitory and activating motifs (indicated with dotted dark blue-light blue arrows).

Indeed, NK cell-mediated anti-tumor responses are also governed by other inhibitory receptors expressed on different immune cells including NK cells, such as T-cell Ig and mucin-containing domain-3 (Tim-3) [18], Lymphocyte Activation Gene 3 (LAG-3) [19] and PD-1 [20] whose ligands are non-MHC class I related molecules (Figure 1). NK cell inhibitory receptors act as immune checkpoints controlling NK cell activation and effector function via engagement of these checkpoints by their ligands on target cells. In the last decade, blockade of these checkpoints offers novel opportunities for cancer immunotherapy [21,22].

Recently, another family of paired receptors that interact with ligands of the family of Nectins and Nectin-like molecules (Necls) has attracted great interest [23,24]. Thus, DNAM-1 (also known as CD226) [25], TACTILE (also known as CD96) [24,26], TIGIT [24,27], and PVRIG [28,29] are receptors that share the same ligands, CD155 (Necl-5) and/or CD112 (Nectin-2), that are also known to exert opposite functions on T and NK cells functions. Whereas DNAM-1 is an activating/co-stimulatory receptor [30] involved in recognition and lysis of tumor cells, TIGIT [27] and PVRIG [28] engagement inhibit NK cell function.

Advances in NK cell-based immunotherapy against cancer rely on the studies of NK cell phenotype (e.g., expression of inhibitory and activating receptors) and lytic capacity (balance between activating and inhibitory signals) that can be regulated by cytokines and on the studies of checkpoint blockade with mAb. In this context, the analysis of the activating receptor DNAM-1 and its paired receptors TIGIT, PVRIG and TACTILE opens new therapeutic possibilities.

## 2. Nectin and Nectin-Like Protein: Expression on Tumor Cells and Their Recognition by NK Cells

Nectins and nectin-like molecules (Necls) are a family of cell adhesion molecules that belong to the immunoglobulin superfamily. They are expressed in many different cell types and mediate both homotypic and heterotypic cell-cell adhesion. Besides their role in cell adhesion, it has been shown that several members of this family can serve as virus receptors (herpesvirus 1 or poliovirus). In addition, some Nectins or Necls can be expressed on cells of the immune system playing an immunoregulatory function by interacting with receptors expressed at the cell surface of other immune cells [31]. As indicated above, some members of this Nectins and Necls family (Nectin-2 and Necl-5) have attracted great interest for their potential use as cancer biomarkers, as they are overexpressed on a variety of tumor cells from different origins, and as potential targets in cancer immunotherapy as they can be recognized by activating and inhibitory paired-receptors expressed on NK cells [23,32,33,34].

### 2.1. CD155 or Necl-5

CD155, also referred as Necl-5, as it belongs to the Necls molecule family, was originally identified as a poliovirus receptor (PVR). It is an immunoglobulin (Ig)-like adhesion molecule, with an important role in cell migration and proliferation [31,35,36]. In the field of tumor immunology, CD155 has gained an importance as it is overexpressed in various human malignancies and is involved in mediating tumor cell invasion and migration [37,38]. As it will be reviewed below, CD155 has an important immunoregulatory functions through its interactions with both co-stimulatory receptor DNAM-1 (CD226) and co-inhibitory receptor TIGIT and TACTILE (CD96) on NK and T cells [39,40,41].

### 2.2. CD112 or Nectin-2

Another member of the Nectin-family molecules is CD112, also termed Nectin-2 or poliovirus receptor-related 2 protein (PVRL2). It is an adhesion molecule that belongs to the Ig gene superfamily and it is involved in the formation of cell junctions [42]. CD112 is closely connected to tumorigenesis, being overexpressed in different types of cancers such as acute myeloid leukemia, multiple myeloma and epithelial cancers [43,44,45]. Besides, it has been reported that CD112 expression is associated with aggressiveness and poor prognosis of gallbladder cancer [46]. CD112 is a ligand for human DNAM-1, and its interaction along with other triggering NK receptors triggers human NK cell-mediated cytotoxicity [39,47]. However, it has been reported that the inhibitory receptors TIGIT and PVRIG also recognize and interact with CD112 leading to inhibition of NK cell-mediated cytotoxicity [29,41].

### 2.3. CD111 (Nectin-1) and CD113 (Nectin-3)

In addition to CD112 and CD155, other Nectins have been recently identified as ligands for TACTILE and TIGIT. Thus, CD111 (Nectin-1 or PVRL1) that was previously described as a receptor for herpes viruses [48] is also a ligand for TACTILE [49], and CD113 (Nectin-3 or PVRL3), that originally was described as a member of Ig-like cell adhesion molecules [50], has recently been identified as a ligand for TIGIT [51].

## 3. Paired Receptors for Nectin and Nectin-Like Proteins

DNAM-1, TIGIT, PVRIG and TACTILE (CD96), constitute a group of Ig superfamily receptors that share the same ligands but show opposite functions. The DNAM-1 activating receptor and the TIGIT/PVRIG/TACTILE inhibitory axis have been shown to be key regulators of anti-tumor immune responses.

### 3.1. DNAM-1

DNAM-1 (also known as CD226) was first discovered as a costimulatory receptor in cytotoxic T cells that was named TLiSA1 for human T lineage-specific activation antigen [52,53]. DNAM-1 is also expressed by NK cells and other immune cells such as monocytes. The extracellular portion of DNAM-1 contains two Ig-like domains and its cytoplasmic tail contains three tyrosine residues. NK cell cytotoxicity is triggered by DNAM-1 cross-linking resulting in Fyn mediated tyrosine phosphorylation [53]. DNAM-1 ligands were identified as PVR (CD155) and Nectin-2 (CD112) [39,54]. DNAM-1 is associated with NK cell education [55]. Thus, according to the expression of DNAM-1 two distinct NK cell subsets have been described in mice, the DNAM1^+^ and DNAM-1^−^ subsets, with different functions. Specifically, DNAM-1^+^ NK cells express high levels of inflammatory cytokines and high proliferative capacity whereas DNAM-1^−^ NK cells produce high levels of chemokines and have greater expression of NK cell receptor genes. DNAM-1^−^ cells differentiate from DNAM-1^+^ NK cells, suggesting that DNAM-1 expression distinguishes the NK cell maturation program status. DNAM-1 expression decreases during NK cells differentiation and the DNAM-1^+^/ DNAM-1^−^ NK cells ratio diminishes from birth [30]. Nevertheless, it is not clear if this model of NK cell maturation applies also to human NK cells. Unlike the mouse, most human NK cells express DNAM-1 and its expression, together with LFA-1, has been linked to NK cell education [56,57]. However, differences in DNAM-1 expression level can represent different maturation status of human NK cells [55]. Down-regulation of DNAM-1 expression on human NK cells has been reported in healthy ageing and in several diseases including cancer [58,59,60,61].

The recognition of CD155 by DNAM-1 potentiates the cytotoxicity of NK cells against a range of tumor cells and has been shown to be critical for tumor immunosurveillance in several murine models [62,63,64], although its significance in immunosurveillance has been controversial as, in some experimental circumstances, the antitumoral effect of DNAM-1 was significant only when the antitumoral response induced by cytokines was analyzed [65].

Cancer cells frequently express high levels of DNAM-1’s ligands and DNAM-1 activation is involved in the killing of cancer cells [33,43,44,47,66]. Indeed, NK cell-mediated lysis of cancer cells was associated with the expression of CD155 in tumor cells from neuroblastoma [67] and ovarian cancer patients [68,69]. Furthermore, the expression of DNAM-1 was decreased in NK cells from acute myeloid leukemia patients and it was negatively correlated with the expression of CD112 in blasts [43] supporting that DNAM-1 downregulation on NK cells in patients with cancer, is a consequence of the tumor burden. Increased expression of DNAM-1’s ligands are induced on multiple myeloma cells after chemotherapy, increasing their susceptibility to NK cell-mediated lysis [70]. NK cells in myelodysplastic syndrome patients show reduced levels of DNAM-1 and NKG2D that correlated with bone marrow blast counts [61]. DNAM-1’s ligands, CD155 and CD112, can be also expressed in some immune cells [71,72]. DNAM-1 cooperates with NKp30 in the lysis of immature dendritic cells expressing DNAM-1 ligands [71]. Activated T cells upregulate CD155 and became susceptible to NK cell-mediated killing, a process that requires the cooperation between DNAM-1 and NKG2D [72].

In the last decade, different experimental models have demonstrated the existence of NK cells with adaptive capacities to remember previous encounters with pathogens such as CMV and to mediate more effective protection against pathogens [73]. It has been shown that DNAM-1 is involved in the generation of NK cells displaying these memory-like functions [74].

The studies of the expression of DNAM-1 in NK cells supports that its interaction with its ligands on tumor cells plays an important role against different types of cancer. However, the discovery in recent years that these ligands are also recognized by inhibitory receptors makes it difficult to interpret the prognostic value of the expression of CD155 and CD112 in tumor cells.

### 3.2. TIGIT

TIGIT, also called VSig9, Vstm3, or WUCAM, was first identified in 2009 as a novel member of the Ig superfamily [41,51,75,76]. TIGIT is an inhibitory receptor that is expressed on immune cells such as effector and memory T cells, regulatory T cells, follicular T cells and NK cells [41,75,76,77,78,79,80]. The cytoplasmic tail contains an immunoreceptor tyrosine-based inhibitory motif (ITIM) and an immunoglobulin tail tyrosine (ITT)-like motif, which are highly conserved between mouse and human [41,51,75,76]. Similar to DNAM-1, TIGIT binds to CD112 and CD155. TIGIT also binds to CD113, another member of the Nectin family (Figure 1) [23,51,81].

It has been suggested that the combined effects of TIGIT signaling through these pathways inhibit cytotoxicity, granule polarization and cytokine secretion in NK cells [41,82,83]. On T cells, the inhibitory effect of TIGIT was initially thought to be indirectly mediated by the induction of tolerogenic dendritic cells [51]. It was later shown that TIGIT can also directly inhibit T cell activation, proliferation and acquisition of effector functions by targeting molecules in the TCR signaling pathway such as CD3ε and PLCγ [75,78].

### 3.3. TACTILE

Human TACTILE, also known as CD96, was discovered in 1992 [84] and it has been investigated to a lesser extent compared to DNAM-1 or TIGIT. It is a member of the Ig superfamily whose expression is almost restricted to T and NK cells [84]. Both human and mouse TACTILE contains an ITIM-like domain in its cytoplasmic portion that mediates inhibitory signaling after engagement with its ligand. Human TACTILE also contains a YXXM motif similar to that of several activating receptors such as NKG2D, however its functional relevance is poorly understood [26]. TACTILE is constitutively expressed on resting human and murine NK cells and mediates NK cell-target cell adhesion by interacting with CD155 [40]. In addition, it has been shown that TACTILE also binds to CD111 [23,49,85] (Figure 1).

In mice, TACTILE binding to CD155 inhibits IFN-γ production [64]. In contrast, human TACTILE seems to have an enhancing effect in NK cell-mediated cytotoxicity [40]. However, Staniestsky et al. failed to confirm these results [41] and in vitro blocking experiments did not demonstrate a role of human TACTILE in NK cell killing of ovarian carcinoma cells [68] or myeloma cell lines [45]. Furthermore, no evidence of a role of TACTILE in the lysis of different tumor cells was found in mice [85].

Despite the results obtained in preclinical models, the role of TACTILE in the modulation of the effector function of human NK cells remains unclear and discrepant results have been reported. The functional discrepancies between human and murine TACTILE relate to the structural differences. The presence of the activating YXXM motif in human but not in mouse TACTILE may result in functional divergences that require further analysis. It has been suggested that human TACTILE exerts inhibitory or activating functions depending on the cell type [26]. Split variants of the second domain of human TACTILE have been described with different binding affinity to CD155 that may also have significant functional relevance [86]. In mouse models, TACTILE acts as a negative regulator of NK cell function and blockade of TACTILE with antibodies, alone or in combination with anti-CTLA-4 or anti-PD-1, promoted antitumor responses [40,64,87]. However, in humans, the role of TACTILE in controlling the activity of NK cells is unclear, since this receptor has motifs of activation and inhibition that could mediate both positive and negative signals in these cells. Additional studies are required to characterize the role of TACTILE in the activation of human NK cells to use it as a checkpoint.

### 3.4. PVRIG

PVRIG, also termed as CD112R, was identified in 2016 [29] as a new inhibitory receptor adding more complexity to this network. PVRIG interacts with CD112 but not with CD155 and represents a novel checkpoint for human T cells [29] and NK cells [28]. PVRIG binds with high affinity to its ligand CD112 on target cells suppressing lymphocyte cytotoxic function. It has been demonstrated that PVRIG and TIGIT are nonredundant inhibitory receptors on CD8^+^ T cells and targeting both pathways enhances antitumor responses in vitro [88].

## 4. DNAM-1 and TIGIT/ PVRIG/TACTILE Axis in the Recognition of Tumor Cells by NK Cells: Blockade of TIGIT and PVRIG Checkpoints in Cancer Immunotherapy

The regulation of NK cells, and also of T cells, by DNAM-1, TIGIT, PVRIG and TACTILE receptors is achieved by complex interactions with their ligands in tumor cells that, depending on the number of inhibitory receptors involved and their binding affinity for CD155 and CD112 on the target cells, will counteract or not the activation signals mediated through the DNAM-1 receptor.

The axis of DNAM-1 and TIGIT/PVRIG/TACTILE, in which shared ligands and different receptor-ligands affinities regulate the immune response, represents a novel checkpoint for improving immune responses against cancer. This balance between inhibitory and activating signals is mediated through cell signaling after the recognition of their ligands that are usually upregulated in tumor cells [24,54,89]. CD155 interacts with DNAM-1, TIGIT and TACTILE and CD112 with DNAM-1, TIGIT and PVRIG. This axis represents a promising target for cancer immunotherapy, but its regulation remains largely unknown.

**Figure 2:**
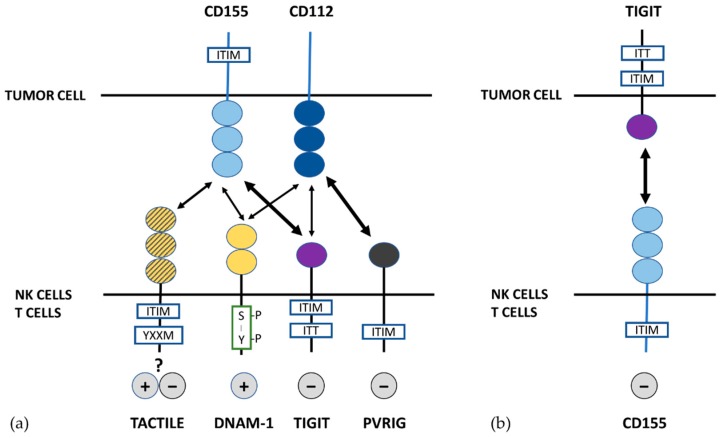
DNAM-1/TIGIT/PVRIG/TACTILE network and its ligands CD155 and CD112. (**a**) DNAM-1, TACTILE, TIGIT and PVRIG on NK and T cells interact with their ligands on tumor cells with different affinities and mediate activating and inhibitory signaling on cytotoxic cells. (**b**) TIGIT expressed on tumor cells may inhibit NK and T cells by interaction with CD155.

Studies on the affinity of these receptors for their ligands (Figure 2a) showed that TIGIT has a higher affinity than DNAM-1 for CD155 and competes for binding to CD155, which interrupts the activation mediated by DNAM-1 and delivers an inhibitory signal to T cells [51]. TACTILE also binds to CD155 with greater affinity than DNAM-1 but lower than TIGIT [87]. In addition, it has been proposed that TIGIT can disrupt DNAM-1 mediated co-stimulation of T cells by interfering with cis-homodimerization of DNAM-1 [90]. Thus, various mechanisms are involved in TIGIT mediated inhibition of T cells [24].

Recently, it has been described the expression of TIGIT in different murine tumor cell lines (colon cancer, breast cancer, melanoma and lung carcinoma) and human colorectal cancer that delivers inhibitory signals to CD8^+^ T cells and NK cells by engaging with CD155 expressed on these immune cells. These results suggest a new pathway for TIGIT-mediated inhibition throughout the interaction of TIGIT expressed in tumor cells with CD155 expressed in immune cells (Figure 2b). These findings support a role of tumor intrinsic TIGIT, helping tumor escape by suppressing the function of NK and CD8^+^ T cells. Interestingly, TIGIT or CD155 blockade with antibodies restores NK and CD8^+^ T cell function further supporting that targeting TIGIT-CD155 interaction could be useful for immunotherapy of cancer [91].

**Figure 3:**
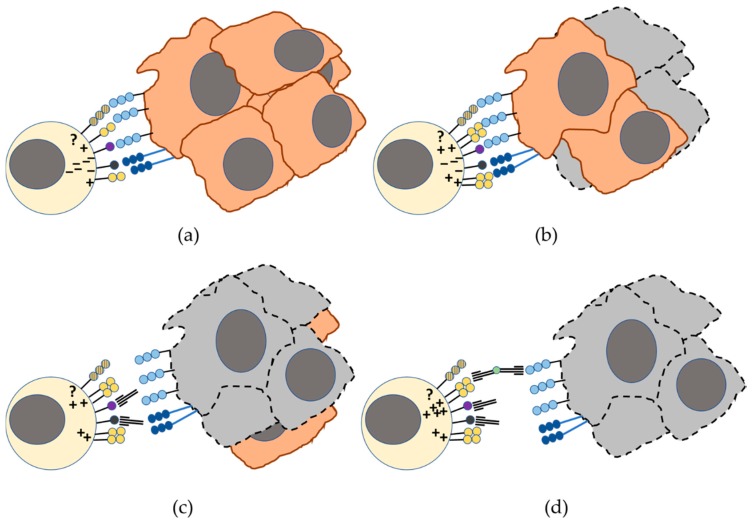
DNAM-1/TIGIT/ PVRIG/TACTILE axis in the recognition of tumor cells by NK cells. (**a**) The activation signals mediated by DNAM-1 (represented in yellow) recognition of its ligands, CD155 (represented in light blue) and CD112 (represented in dark blue), can be counteracted by inhibitory signaling through TIGIT and PVRIG inhibitory receptors (represented in purple and dark grey). In humans, the role of TACTILE (represented in shaded-yellow) in this balance requires a more detailed analysis. (**b**) Overexpression of DNAM-1 can induce lysis of some tumor cells but fails to eliminate the tumor. (**c**) TIGIT or PVRIG checkpoint blockade improves the responses mediated by DNAM-1 contributing to tumor elimination. (**d**) The combination of one or two checkpoint mAbs with co-stimulatory bispecific mAb (e.g., anti-DNAM-1 × anti-CD155) may result in better antitumor immune responses by increasing the interaction of DNAM-1 with its ligands. Cells surrounded by dotted lines represent dead cells.

The effect of immunotherapy with checkpoint blockade has been shown to induce long-lasting responses in some patients with solid and hematological tumors. However, blocking a single receptor as monotherapy will unlikely elicit an effective immune response whereas combinatorial approaches should be tested to improve immune responses against cancer (Figure 3). In a similar way the recognition of CD112 on tumor cells by PVRIG expressed on T and NK cell is considered as a novel checkpoint [29]. On the contrary, although TACTILE has one intracellular inhibitory motif and transmit inhibitory signals in some experimental conditions, it also has an YxxM activating motif. Therefore, this receptor has not been considered as a therapeutic target in the immunotherapy of cancer in humans and until now anti-TACTILE antibodies have not been considered for evaluation in clinical trials. NK cell-based immunotherapies will also benefit from approaches that reverse the immunosuppressive tumor microenvironment. In addition, chimeric antigen receptor (CAR) technology can also be used to generate novel CAR-NK cells for adoptive therapies in order to overcome the NK cell suppression by inhibitory receptors [92]. CAR engineered NK cell lines against several antigens for solid tumors that specifically lyse target cells in vitro and with efficiency in preclinical studies have been generated, as reviewed by Nayyar et al. [93].

### 4.1. Anti-TIGIT mAbs in Clinical Trials for Checkpoint Cancer Immunotherapy

TIGIT has emerged as a novel inhibitory receptor that can be targeted by mAb and represents a new checkpoint for the development of immunotherapy strategies against cancer. In mouse models, TIGIT in combination with PD-1 blockade has been shown to act synergistically to enhance CD8^+^ T cell function [90]. TIGIT blockade has been shown to enhance NK cell functional capacity [27]. Anti-TIGIT mAb with different isotypes and mutant forms designed to eliminate Fc-FcγR interactions, that have been shown to be deleterious in blocking PD-1 [94], have been developed for their potential as checkpoint inhibitors. Many of the clinical trials designed to analyze the significance of anti-TIGIT antibodies in cancer immunotherapy use them in combination with antibodies blocking other checkpoints, in particular PD-1/PD-1L interactions. Expression of PD-1 in NK cells is restricted to a subset of functionally exhausted NK cells characterized for being CD56dim NKG2A–KIR+CD57+ [20]. In a recent study in murine solid tumors, the presence of NK cells was shown to be critical for the therapeutic effects of blockade of TIGIT checkpoints or the combined blockade of TIGIT and PD-1/PD-1L checkpoints [27], supporting the relevance of the TIGIT signaling pathway in the immune escape of tumor cells. In addition, these results also support that targeting different pathways by using combined anti-tumor immunotherapy strategies can be critical to revert NK cell defective capacity to eliminate cancer cells (reviewed in [93]).

The characteristics of phase 1 or phase 2 clinical trials using anti-TIGIT mAbs alone or in combination with different anti-PD-1/PD-1L mAbs, including the recruitment status (as in April 2019) and the estimated completion date, are summarized below and in Table 1.

Etigilimab (OMP-313M32) is an anti-TIGIT IgG1 mAb developed by OncoMed Pharmaceuticals. It is under study in a phase 1 clinical trial (NCT031119428) in solid tumors as monotherapy or in combination with Nivolumab (anti-PD-1).

Tiragolumab (also known as MTIG7192A and RG6058), is an anti-TIGIT mAb developed by Genentech/Roche, is a fully human IgG1 antibody that binds to human TIGIT. A phase 2 clinical trial (NCT03563716) is evaluating its use alone or in combination with Atezolizumab (anti-PD-1L) in chemotherapy-naïve patients with locally advanced or metastatic non-small cell lung cancer.

AB154, an IgG4 anti-TIGIT mAb developed by Arcus Bioscience, is included in a phase 1 clinical trial (NCT03628677) to evaluate the safety, pharmacokinetics, pharmacodynamics and preliminary efficacy in advanced solid tumors as monotherapy or combined with AB122 (anti-PD-1). 

MK-7684 (IgG1) anti-TIGIT mAb was developed by Merck Sharp & Dohme and it is being evaluated in phase 1 in patients with advanced solid tumors either as monotherapy or in combination with Pembrolizumab (anti-PD-1).

In addition, two mAbs with mutated Fc to avoid binding to FcγR are under study:

BMS-986207 (anti-TIGIT IgG1, FcγR-null) from Bristol-Myers Squibb is evaluated in a phase 1/2 clinical trial (NCT02913313), as monotherapy or in combination with Nivolumab (anti-PD-1) in advanced solid tumors.

ASP8374 (anti-TIGIT IgG1, FcγR-null) developed by Astellas Pharma; Potenza Therapeutics. It leads to the activation of cytotoxic T-lymphocyte CTL mediated response against cancer cells. There are currently two clinical trials with participants with locally advanced (unresectable) or metastatic solid tumor malignancies, a phase 1 clinical trial (NCT03260322) evaluating the tolerability and safety profile of ASP8374 when administered as a single agent and in combination with Pembrolizumab and a phase 1 study (NCT03945253) to evaluate the tolerability and safety profile and to characterize the pharmacokinetic profile of ASP8374.

### 4.2. PVRIG as a Novel Checkpoint for Cancer Immunotherapy

PVRIG is a recently identified inhibitory receptor that interacts with CD112 competing with DNAM-1. Blocking PVRIG interaction with CD112 with antibodies enhanced T cell stimulation [29]. In humans, blockade of PVRIG and TIGIT has been shown to enhance trastuzumab-mediated NK cell response against breast cancer [28]. Antibody dependent cell cytotoxicity (ADCC) mediated by NK cells is one of the major mechanisms of action for trastuzumab against Her2 positive breast cancer. It has been shown that, among the possible strategies to enhance trastuzumab-mediated ADCC by NK cells, blockade of PVRIG enhances trastuzumab-triggered anti-breast cancer response [28], therefore supporting that this receptor plays a suppressive function on NK cells. Based on these evidences, PVRIG can be considered a therapeutic target and its blockade in vivo could imply a novel approach to improve trastuzumab efficacy in human breast cancer. Up to now there is only one anti-PVRIG antibody used in a clinical trial for cancer immunotherapy, COM701, that is being tested in a phase 1 clinical trial (NCT03667716) to evaluate its safety, tolerability and preliminary clinical activity, as monotherapy or in combination with Nivolumab, in patients with advanced solid tumors including non-small cell lung cancer, ovarian, breast and endometrial cancer.

## 5. Conclusions

Considering the importance of the immune regulatory network mediated by DNAM-1, TIGIT, TACTILE and PVRIG that involve Nectin and Nectin-like ligands, it is of interest to understand the mechanisms governing this axis and the factors contributing to the inhibitory/activating outcome after engagement. NK cells are now being considered a promising therapeutic target for cancer immunotherapy, due to its peculiar ability to kill diverse tumor cells. Thus, understanding of the input of immune checkpoints in the regulation of NK cell functions and determining the contribution of NK cells to the clinical interest of blockade of these checkpoints will open the door for the discovery of new therapies for different types of cancers.

## Figures and Tables

**Table 1:** Clinical trials based on checkpoint blockade using mAbs against TIGIT.

Clinical Trial Identifier	Condition or Disease	α-TIGIT mAb	Intervention	Phase	Recruitment Status April 2019	Estimated Study Completion Date
NCT03119428	Advanced Cancer, Metastatic Cancer	Etiligimab (OMP-313M32)	OMP-313M32; OMP-313M32 + Nivolumab	1	Active, not recruiting	October, 2019
NCT03563716	Non-small Cell Lung Cancer	Tiragolumab (MTIG7192A)	MTIG7192A Atezolizumab; Placebo + Atezolizumab	2	Active, not recruiting	February, 2021
NCT03628677	Advanced solid tumors	AB154	AB154; AB154 + anti-PD1 (AB122)	1	Recruiting	February, 2020
NCT02964013	Advanced solid tumors	MK-7684	MK-7684; MK-7684+ Pembrolizumab	1	Recruiting	June, 2022
NCT02913313	Advanced solid tumors	BMS-986207	BMS-986207; BMS-986207 + anti-PD1	1/2	Recruiting	December, 2022
NCT03260322	Advanced solid tumors	ASP8374	ASP8374; ASP8374+ Pembrolizumab	1	Recruiting	July, 2021
NCT03945253	Advanced solid tumors	ASP8374	ASP8374	1	Not yet recruiting	December, 2022
